# Assessing the Landscape Ecological Risks of Land-Use Change

**DOI:** 10.3390/ijerph192113945

**Published:** 2022-10-27

**Authors:** He Gao, Wei Song

**Affiliations:** 1 Key Laboratory of Land Surface Pattern and Simulation, Institute of Geographic Sciences and Natural Resources Research, Chinese Academy of Sciences, Beijing 100101, China; 2 School of Geosciences, Yangtze University, Wuhan 430100, China; 3 Hebei Collaborative Innovation Center for Urban-Rural Integration Development, Shijiazhuang 050061, China

**Keywords:** land-use change, landscape pattern, ecological risk, theoretical framework of ecological risk transformation of land-use change, ecological fragile area, Zhangjiachuan county, China

## Abstract

In recent years, a changing global climate and the continuous expansion of the intensity and scope of human activities have led to regional differentiation in the surface landscape. This has caused numerous ecological risks under multiple pressure sources, gradually becoming an important factor restricting the sustainable development of economic and social health. With the continuous development of the social economy, land use and associated ecological risks will inevitably change. According to the forest transformation theory and the environmental Kuznets curve, we put forward the theoretical framework of ecological risk transformation of land-use change and took Zhangjiachuan County (China) as an example to verify it. Therefore, on the basis of Landsat satellite data, this paper used landscape structures to calculate an ecological risk index, and evaluated the ecological risk of land-use changes through pattern index analyses. The results show that, from 2000 to 2020, the ecological risk index of land-use change in Zhangjiachuan County exhibited an increasing and then decreasing trend, showing an overall “inverted U-shaped” trend of change consistent with the transformation theoretical framework of ecological risks of land use change. Secondly, in terms of patterns, the ecological risk of land-use change in Zhangjiachuan County showed a distribution feature of high in the west and low in the east. In 2000, high-risk areas were mainly concentrated in the central and northern areas, while low-risk areas were mainly concentrated in the eastern areas. From 2000 to 2015, the medium-risk areas expanded to the west and midwest, and the geographic centers of the risk areas were slightly offset. From 2015 to 2020, the overall pattern of ecological risk areas was basically the same as that of the previous stage, but the medium-risk areas were slightly reduced. In terms of quantity, from 2000 to 2015, the areas of the lowest risk level and low risk level decreased, while the areas of medium risk level, high risk level, and the highest risk level increased; from 2015 to 2020, the areas of the lowest risk level and low risk level increased, and the areas of medium risk level, high risk level, and highest risk level decreased. Lastly, the spatial aggregation of ecological risks in Zhangjiachuan County weakened slightly from 2000 to 2005, gradually increased from 2005 to 2015, and then slightly weakened from 2015 to 2020.

## 1. Introduction

Over the centuries, the multiple effects of human activities and natural succession have led to dramatic changes in land use and global ecology, inevitably triggering various conflicts between economic growth and sustainable development [1,2]. The ecological risks caused by land-use changes are currently among the hottest issues of concern in developed and developing countries worldwide [3]. The extensive influence of land-use changes and ecological changes has led to changes in ecosystem structures and landscape patterns, leading to a large number of ecological problems, such as ecological function degradation, soil erosion, land desertification, environmental pollution, and biodiversity reduction [4,5]. The ecological environment is constantly affected by these problems, greatly increasing the risk posed to ecosystems [6] and seriously threatening human well-being [5,7,8]. Assessing the ecological risks of land-use changes is critical for establishing early warning systems of ecological risks, with an accurate and effective control of ecological risks, and it is of great significance to the formulation of regional ecological protection policies and for adopting ecological protection measures.

Risk assessment began in 1980 and initially consisted of toxicology research on chemical pollutants and risk research on human health. It then became used as a kind of management tool to assess multiple chemical pollutants and various events that may cause environmental pollution and was finally extended to consider the assessment of ecological risks caused by human activities. The term “ecological risk assessment” was first introduced by the US Environmental Protection Agency (EPA), which defined ecological risk as the possibility of exposure to adverse ecological effects caused by or likely to result from one or more stressors [9], and the framework was subsequently expanded and revised to form the basic guide for the current risk assessment [10]. Since 1990, in the context of increasingly prominent ecological problems, the focus of risk assessments has gradually shifted from human health assessments to ecological risk assessments [11], and risk receptors have expanded to populations, communities, and whole ecosystems. From the late 1990s to the beginning of the 21st century, with the continuous improvement and maturation of ecological risk assessment systems, the field of ecological risk assessment gradually expanded and entered the stage of regional ecological risk assessments, and the risk receptors extended to the watershed and regional landscape scales.

Landscape ecological risk assessment is an important branch of ecological risk assessment [12], which tends to quantitatively identify and directly assess ecological risks from the perspective of spatial landscape patterns caused by land-use changes. Since land use is regarded as a comprehensive reflection of the direct impact of human economic and social activities on surface resources and natural environment [13], the spatiotemporal heterogeneity of land use is affected by regional terrains and geomorphic features closely related to landscape patterns and landscape ecological risks [14]. At present, the research on the ecological risks of land-use changes has mainly focused on analyzing long-term historical evolution characteristics [15,16], revealing the driving mechanisms of ecological risks caused by land-use changes [17], and simulating future evolution trends [18,19]. On the one hand, through the analysis of the long-term historical evolution characteristics of the ecological risks of land-use changes in a region, we can obtain reliable evidence for the long-term changes of ecosystems in the region, reveal its evolution trajectory and mechanisms, and provide key scientific information for the formulation of regional ecological restoration paths and objectives. For example, Recanatesi et al. [20] analyzed the changing characteristics of soil vulnerability and landscape degradation and their spatial distribution during the period from 1960 to 2010 in Italian agricultural and forestry areas using four thematic indicators of environmentally sensitive areas and the comprehensive index of desertification risk. Krajewski et al. [21] used the landscape change index to analyze the driving mechanisms of forest resource changes and forest transformation in landscape parks on the basis of 140 years (1863–2013) of map data. On the other hand, ecological risk simulations of regional land-use changes allow us to predict the possible impact or harm caused by human activities on the ecosystem and formulate ecological protection and risk prevention policies, which are of great significance for promoting regional sustainable development. For example, Li et al. [22] used the conversion of land use and its effects at a small region extent (CLUE-S) model to simulate and analyze the future land-use changes of the Luanhe River basin from 2010 to 2030 under three scenarios of trend, rapid economic growth, and ecological security, and then assessed the spatial distribution characteristics of the landscape ecological risks caused by it.

With the rapid development of geographical information system (GIS) and remote sensing (RS) technologies and the wide application of landscape ecology theories and methods [23], landscape ecological risk assessment based on land-use changes has become one of the most effective methods in ecological risk assessment [24]. At present, the ecological risk assessments have mainly focused on cities, river basins, coastal areas, administrative regions, and nature reserves. The urban populations of cities are excessively dense, and their spatial structure expands rapidly, with various land-use types being frequently transformed into each other, and ecological problems being increasingly prominent [25,26]. The watershed area ecology is fragile, and the natural endowment conditions such as water resources are poor. The ecological improvement and deterioration of landscapes coexist, with the overall deterioration trend being greater than the improvement one, and the degree of ecological deterioration of a landscape is constantly increasing [27,28]. In the process of development in coastal areas, the unrestricted urban expansion during the early stages of economic development has led to landscape fragmentation, and the overall ecological risk has increased, becoming more spatially clustered. With the suppression of disorderly expansion, the overall ecological risk decreases [29,30]. During the early stages of the development planning of administrative regions, the purpose of regional economic development is usually achieved by sacrificing agricultural lands. During this period, land use changes drastically, resulting in increased ecological risks. Subsequently, due to the gradual enhancement of residents’ awareness of environmental protection and the implementation of targeted ecological prevention and control and protection policies, ecological problems caused by human activities have been alleviated and ecological risks have been reduced [31,32,33]. Nature reserves can be divided into core areas, buffer zones, and experimental zones according to their functions. Human activities are common in the experimental areas, the ecological risk of land-use changes is the most serious, and the land-use changes in the buffer zone and the core area are relatively stable. Therefore, the ecological risk of nature reserves is greatest in the experimental areas, followed by the buffer areas, and finally the core areas [34,35].

Compared with traditional ecological risks, the landscape ecological risk assessment pays more attention to spatial heterogeneity, and its key lies in establishing an evaluation system and selecting appropriate indicators. At present, there are two widely used methods: one is the ecological risk assessment system based on the “pressure–receptor–response” model and failure mechanism [25]. The ecological risk assessment system consists of identifying the risk source intensity, receptor exposure, and risk effect. The evaluation method is a comprehensive relative risk model (RRM) [36]. The evaluation system focused on stressors and habitats of concern in the study area. For example, Muditha and Heenkenda et al. [37,38] used this evaluation system to rank and classify the stressors and habitats within a region and modeled the interaction between the two through exposure and effect filters, revealing the spatial and temporal distribution of ecological risk in ports. However, this model is only suitable for large-scale areas that need to focus on multiple stressors and is often used to assess the ecological risks of a specific stressor or disturbance source, which has certain limitations. The second approach uses the deviation from the optimal mode as a risk source, evaluating the ecological risk, and regarding the whole system as a receptor. The landscape pattern index is commonly used to assess the ecological risks of the study subjects as a function of the ecological changes throughout a region, and the most representative method is the landscape loss model. Shi et al. [39] used the Markov model and landscape index analysis to construct an ecological risk assessment model, which revealed that the ecological risks in Huaibei, a typical resource-based city in China, were affected by land-use changes. In addition to assessing the landscape ecological risks alone, some scholars have also studied the ecosystem services associated with landscape ecological risks. For example, Gong et al. [40] revealed the spatiotemporal changes in the grain yield of cultivated land, carbon storage, water yield, biodiversity index, and ecological risks in the Bailongjiang River basin of China by introducing ecosystem services and landscape ecological risks into the formulation of ecological policies and governance of ecological problems. It is difficult to obtain RRM assessment data and completely uniform assessment criteria during the comparative analysis of different time series in the same study region. Therefore, with the support of a landscape ecology theory, a landscape loss model based on land-use changes can both quantitatively describe the landscape structure [41,42] and explain the evolutionary mechanisms of landscape ecological risks from the perspective of spatial landscape pattern changes. This model becomes an important tool to analyze and reveal the spatial and temporal characteristics of landscape ecological risks.

The ecological risk of land-use change has obvious stages. Analyzing and studying the evolution characteristics of the ecological risks of land-use changes on medium and long timescales can provide a scientific and reasonable basis for the planning of regional ecological protection schemes. Earlier research on this topic mainly focused on the construction of ecological risk models and spatial analysis [29], and relatively little theoretical analysis has been conducted on the ecological risks of land-use changes on medium to long timescales. We previously studied the ecological risk changes of land-use changes in other countries and found that there is indeed a close relationship between the pattern evolution of ecological risks and land-use changes. Therefore, according to the theories of forest transformation and an environmental Kuznets curve, this paper reconstructed a theoretical framework for the ecological risk transformation of land-use changes and put forward a theoretical hypothesis. Considering Zhangjiachuan County, Gansu Province, China as the verification case area, this paper constructs an ecological risk assessment model through landscape disturbance and landscape vulnerability indices by taking into account land-use changes in order to comprehensively reveal the evolution characteristics of the overall spatial and temporal patterns of ecological risks in Zhangjiachuan County. Specifically, the research objectives of this paper were to (1) analyze the spatiotemporal land-use change characteristics in Zhangjiachuan County from 2000 to 2020, (2) reveal the spatiotemporal evolution patterns of the ecological risks of land-use changes, and (3) study the dynamic characteristics of ecological risks at the landscape level through spatial autocorrelation analyses. The remainder of this paper is arranged as follows: in Section 2 and Section 3, brief descriptions of the study area, datasets, and methods are provided; Section 4 introduces the results of land use and landscape ecological risk changes in Zhangjiachuan County; the discussion and conclusions are then provided in Section 5 and Section 6, respectively.

## 2. Overview of the Study Area and Data Sources

### 2.1. Overview of the Study Area

**Figure 1:**
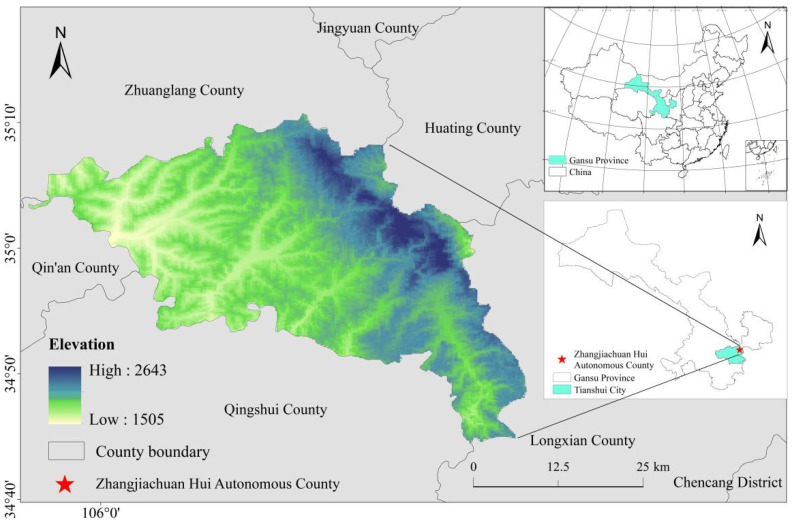
Geographical location of Zhangjiachuan County.

Zhangjiachuan County (105°54′–106°35′, 34°44′–35°11′) is located in the southeast of the Gansu Province, the northeast of Tianshui City, at the foot of the west side of Longshan Mountain. Its northern part is linked with Zhuanglang County and Huaxiang County in Pingliang City. To the south is Tianshui City, Qingshui County, and it is adjacent to Qin’an County, Tianshui City to the west, and Long County, Shaanxi Province to the east. Zhangjiachuan County is located in the transition zone of Liupanshan trough and Longxi Loess Plateau, belonging to the loess hilly and gully region in the middle reaches of the Yellow River. The terrain is uneven in most areas, with alternating ridges and ravines, small plots, steep slopes, and the terrain slopes from northeast to southwest. The county is 62 km long from east to west and 48 km wide from north to south, with a total area of 1311.8 km^2^ (Figure 1).

Zhangjiachuan County is a traditional dry farming county, with deep mountains and fragmented terrains. The average elevation is 2011.4 m, with the highest point at 2659 m and the lowest at 1486 m. The climate is warm temperate semi-humid, with an average temperature of 7.6 °C, highs of 31.7 °C, and lows of 20.6 °C. The annual average rainfall is 599.8 mm, and the appropriate amount of precipitation varies greatly in time and space, with the months of July, August, and September accounting for 59.3% of the annual rainfall. The rainfall in Zhangjiachuan County falls mostly through heavy rainstorms which last for a long time and are of high intensity, which can easily cause soil erosion. In recent years, with the enhancement of human activities, the natural vegetation has declined sharply with soil erosion becoming serious, and the land has been seriously degraded. In addition, the geographical environment in the study area is complex, and the climate is volatile, leading to frequent natural disasters such as droughts, hails, rainstorms, and floods. Overall, the ecosystem in the study area is relatively fragile and the sustainable development of the ecosystem has become threatened [43].

### 2.2. Data Source

This paper used two types of data to conduct a land-use classification and landscape ecological risk assessment:

(1) Landsat image data. The primary data source used in this study was Landsat satellite images. The satellite images included Landsat TM (2000–2011), Landsat ETM + (2012), and Landsat OLI (2013–2020). The images were filtered using a series of functions in the Google Earth engine (GEE) filter, with a time standard of April–September per year and a cloud coverage standard of less than 10%. The available remote sensing images were generated following cloud removal, image mosaicking, and cropping processing. The specific data sources are shown in Table 1.
(2) Other data. Using the administrative boundary data of the National Catalog Service for Geographic Information [44], we combined the 30 m resolution digital elevation model (DEM) data of the geospatial data cloud platform to serve as the classification basis [45]. The analyses were performed using Google historical image data (2000–2020) from 91 Bitmap Assistant [46] with a resolution of 0.52 m.

## 3. Research Method

**Figure 2:**
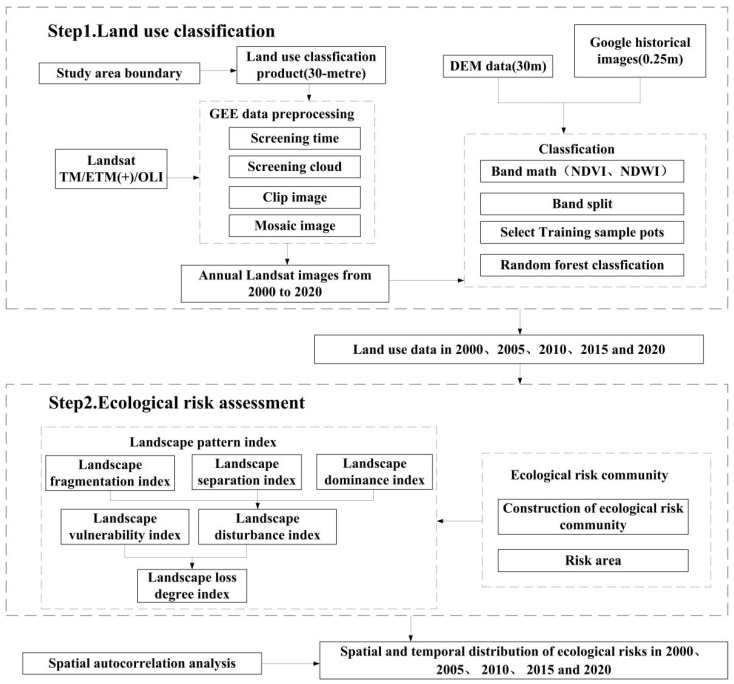
Ecological risk assessment process of land-use change.

The main technical steps to assess the landscape ecological risk in Zhangjiachuan County are shown in Figure 2. First, the images were preprocessed through the GEE online editor, and the image data from 2000 to 2020 were classified using the random forest classifier. Then, the landscape ecological risks were evaluated, and the spatial and temporal change characteristics were explored. Finally, the spatial autocorrelation of the ecological risk index was analyzed using the Moran’s I index and local spatial autocorrelation analysis methods.

### 3.1. Theoretical Framework of the Ecological Risk Transformation of Land-Use Changes

The main basic theories of the ecological risk transformation theory framework of land-use changes include the forest transformation theory and environmental Kuznets curve. Among them, Mather [47] divided the forest area into two stages of a “U-shaped” change process, from a reduction to an increase. According to the Kuznets curve proposed by Simon, Panayotou [48] divided the change relationship between environmental quality and per capita income into two stages, namely, the “inverted U-shaped” change process, in which environmental quality first increases and then decreases with the per capita income. In the process of forest transformation, the process of abandoned logging land being reclaimed and developed into agricultural lands by farmers can reflect the change in land use, while the economic, social, and ecological issues, among others, which are brought about by social and economic development, can also be fed back to land use. Therefore, Mather’s forest transformation theory and the Panayotou environmental Kuznets curve are also applicable for the ecological risk change process of land-use changes and show the characteristics of stages.

**Figure 3:**
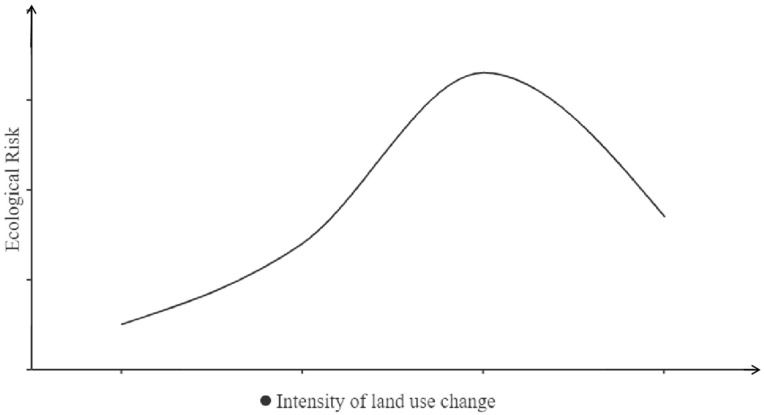
Relationship curve showing the ecological risk of land-use change.

The root of ecological risk change lies in the change in land use, and its essence depends on the mutual feedback of land-use changes and ecological risks under transmission mechanisms. It not only reflects the consistent change characteristics of the similarity attribute and the transformation theory in different social development stages, but also exhibits a trend of inconsistent change rates in different development stages under the combined effect of natural factors and human interferences. Lastly, the ecological risk, accompanied by the land-use changes, presents an “inverted U-shaped” phase change difference, forming a “land-use change ecological risk” relationship curve (Figure 3).

Ecological risks present an “inverted U-shaped” change trend with land-use changes. Land-use changes are dynamic and characterized by large interannual changes and inconsistent change rates. Therefore, the change rate of the ecological risks of land-use changes fluctuates at different stages. At the initial stage of social development when the economy is in its infancy, the process of urbanization is slow. Driven by the national macro policies, the construction of large-scale development zones has led to an increase in the area of urban and rural land and industrial and mining construction land. At the same time, the incentive policies issued in urban construction, land management, and other major infrastructure construction have led to an increase in construction land. In order to meet the demands for the expansion of construction land, the occupation of cultivated and ecologically significant lands has gradually increased, resulting in the fragmentation of ecological landscapes, the destruction of vegetation and biological natural habitats to a certain extent, and the continuous reduction in the ecological carrying capacity and environmental capacity, whilst affecting the ecological quality.

In the process of land use, human beings pursue a highly efficient economic society, accelerate the demand for land resources, and ignore the nonrenewable nature of land resources. In the middle period of social development, with accelerated economic development, industrialization and urbanization promote each other; the proportion of nonagricultural industries and nonagricultural populations increases sharply, urban spaces begin to spread to the surrounding areas, and cultivated and ecological lands are largely occupied, resulting in a sharp deterioration of surface natural conditions, a sharp decline in green space areas, and a large consumption of natural resources, thereby compromising the productivity and production functions of certain ecosystems. On the other hand, the remote coupling theory, involving the relaxation of population mobility controls and population migration, typically represented by migrant workers, promotes land-use changes in different ways. Population losses and village abandonment have become a common phenomenon, accompanied by the marginalization and even abandonment of agricultural lands, causing further damage to the landscape connectivity. During this period, land use changed dramatically, causing a sharp increase in ecological risks.

Following economic maturation, the ecological risk reaches its peak, and the increasingly serious ecological problems have gradually aroused great attention of the country. According to the “win–win sustainable development strategy” demonstrated by Gao [49], the ecological risks caused by land-use changes can be mitigated through the combined effects of economic and ecological benefits. The intensification of land use should be strengthened by fully considering the bearing capacity of environment, resources, and ecology. It is necessary to divide the area according to the nature of the city, environmental conditions, and functions, reasonably adjust the industrial structure and construction layout, make the evolution pattern of construction land more compact, and continuously reduce its fragmentation and the separation of individual patches. The intensification of construction lands triggered during this social development stage is beneficial to the improvement of the regional ecological stability. At the same time, the land ecological project, the project of returning cultivated land to forest and grass, land reclamation construction, and regional environmental management, such as water and soil loss management, can be carried out to classify and use scarce land resources, so that forest coverage and species richness can gradually increase. Furthermore, the quantity and quality of grass, wetland, and other ecological land spaces can be improved and abandoned lands can be reused, thereby increasing the connectivity of the landscape. At this stage of development, land use intensity tends to slow down, ecological damage is improved, and ecological risks are gradually reduced.

### 3.2. Land-Use Classification

The image interpretation was mainly carried out in GEE. Following the spatiotemporal filtering and cloud removal processing of the Landsat images, functional methods were used to stitch the images, and the classification accuracy of vegetation and buildings was improved by adding the normalized difference vegetation index (NDVI) and normalized difference building index (NDBI) together. The NDVI values were calculated from the crop growth period (screening time from April to September). The NDVI and NDBI indices were calculated using the following formulas [50]:

(1) NDVI=(NIR−R)/(NIR+R),

(2) NDBI=(SWIR−NIR)/(SWIR+NIR),

where *NIR*, *R*, and *SWIR* are the surface reflectivity of the near-infrared, red-band, and short-wave infrared, respectively.

The B2, B3, B4, B5, B6, and B7 bands of Landsat OLI images (six bands corresponding to Landsat TM/ETM+ images) were combined, cut, and spliced, and six auxiliary classification spectral indices (Table 2) were combined to improve the classification accuracy. The quality of the training samples directly affected the final classification results. This paper combined digital elevation model (DEM) data and Google historical images to provide a visual interpretation for drawing training samples [51,52,53]. According to the land-use classification standard and the actual situation of the research area, the land-use types in Zhangjiachuan County were divided into five categories: cultivated land (623 sample rectangles), forest (503 sample rectangles), grass (516 sample rectangles), construction land (218 sample rectangles), and water areas (183 sample rectangles). In order to ensure the accuracy of the land use classification results, the Landsat image set and Google historical imagery were superimposed and compared to extract the land parcels that have remained unchanged for several years. Combined with the random points generated by Arc GIS, these random points were used as a reference for the selection of the samples, so as to ensure that the selected year-by-year classified samples were evenly distributed in the entire study area. The time iteration and cyclic classification were performed in the GEE program using a combination of a supervised classification and manual visual interpretation to obtain land-use data for 2000, 2005, 2010, 2015, and 2020, and the classification accuracy was verified using Google historical imagery.

A random forest classifier was selected to plot the land-use situation. The random forest method is a comprehensive multi-decision tree-based comprehensive classifier trained and predicted by Breman which consists of a decision-tree classification using the bagging strategy and in-house algorithms on a GEE platform [51]. Due to its high classification accuracy, relatively robust performance, the ability to include more variables, fast prediction speed, good multisource remote sensing data processing capabilities, etc., it has been widely used in land-use classification [60]. The images in the study area were classified by running a random forest in the GEE, using 70% of the data as training samples and 30% as validation samples. To improve the accuracy of the classification results, two parameters were set for the random forest classifier; the number of decision trees was set to 100, and the number of feature variables was the square root of the total number of feature variables [61,62].

The selected validation samples were used as true values to analyze the accuracy of land-use classification from 2000 to 2020. The overall accuracy and Kappa coefficient could be calculated using the confusion matrix tool in the GEE platform. The total classification accuracy is equal to the total number of correctly classified pixels divided by the total number of pixels. The Kappa coefficient and overall accuracy can be measured according to the following equations [63]:

(3) $$P_{e}=\frac{\sum_{i=1}^{m}a_{i}*b_{i}}{n^{2}},$$

(4) $$k=\frac{p_{o}-p_{e}}{1-p_{e}},$$

where *p_o_* is the overall classification accuracy, *a_i_* is the true number of samples for each land-use type, *b_i_* is the number of predicted samples for each land-use type, *m* is the total number of land-use types, *n* is the total number of samples, and *k* is the Kappa coefficient. The precision assessment showed that the overall accuracies of land-use classification in 2000, 2005, 2010, 2015, and 2020, were 87.18%, 85.50%, 88.58%, 91.29%, and 89.05%, respectively, meeting the overall requirements of landscape research [64].

### 3.3. Construction of an Ecological Risk Cell

**Figure 4:**
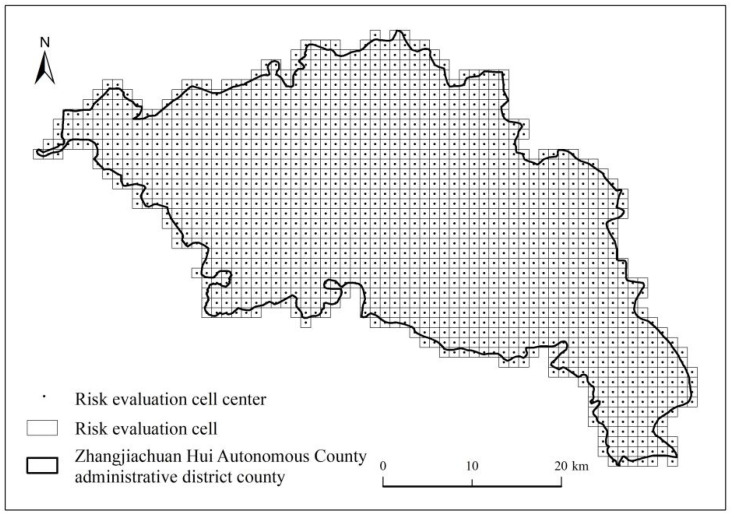
Ecological risk assessment unit of Zhangjiachuan County.

To fully demonstrate the spatial differentiation of landscape indicators and ecological risks in Zhangjiachuan County, this study combined the actual situation and divided it into 1378 ecological risk assessment units consisting of squares with a side length of 0.1 km (Figure 4). The ecological risk index was calculated in each cell, and the results were assigned to the central point of the assessment unit [34].

### 3.4. Construction of Landscape Ecological Risk Indices

The extent of an ecological risk depends on the strength of the regional ecosystem subjected to external interferences and the size of the internal resistance. Different landscape types have different roles in protecting species, maintaining biodiversity, improving the overall structure and function, and promoting the natural succession of landscape structures; moreover, different landscape types have different resistances to external interferences [65]. In this paper, the ecological risk index was calculated to estimate the ecological risks of Zhangjiachuan County in 2000, 2005, 2010, 2015, and 2020, and to reflect on the relationship between the landscape patterns of land-use changes and the ecological risks associated with those. The calculation formula was as follows [66]:

(5) $${ERI}_{i}=\sum_{i=1}^{N}\frac{A_{ki}}{A_{k}}R_{i},$$

where *ERI_i_* is the ecological risk index of the *i*-th risk cell, *A_ki_* is the area of class-*i* landscape of the *k*-th risk cell, *A_k_* is the area of the *k*-th risk cell, and *R_i_* is the landscape loss index [67] of class-i landscape.

(6) $$R_{i}=E_{i}*F_{i},$$

where *F_i_* is the ecological fragility index, which refers to the fragility of the ecosystem under a strong external disturbance of human beings. A smaller fragility denotes a greater resistance and less risk to the ecosystem. However, the variability of different landscape types in response to external interference is related to the stage of natural succession applied [66,68]. The ecological risk orders of different land-use types from low to high were construction lands, forests, grasses, cultivated lands, and water areas. After normalization, the *F_i_* values for the five land-use types were 0, 0.25, 0.5, 0.75, and 1, respectively [69,70,71,72]. *S_i_* is the index of landscape disturbance degree for the category *i* land-use type. The calculation formula is as follows [73]:

(7) $$E_{i}=aC_{i}+bN_{i}+cD_{i},$$

where *C_i_* is the landscape fragmentation degree, *N_i_* is the landscape separation degree, *D_i_* is the landscape dominance degree, and *a*, *b*, and *c* reflect the influence of human interference on the ecosystem, representing the weights of *C_i_, N_i_*, and *D_i_*, respectively; *a* + *b* + *c* = 1.

*C_i_* reflects the changes in the landscape structure, function, and ecological processes; its calculation method is as follows [32]:

(8) $$C_{i}=\frac{n_{i}}{A_{i}},$$

where *n_i_* is the number of patches of landscape type *i*, and *A_i_* is the total area of landscape type *i.*

*N_i_* is the degree of separation of individual patches in a given landscape, expressed as follows [74]:

(9) $$N_{i}=\frac{A}{2A_{i}}\sqrt{\frac{n_{i}}{A}},$$

where *A* is the total landscape area, *n_i_* is the number of patches of landscape type *i*, and *A_i_* is the total area of landscape type *i*.

*D_i_* describes the advantages of patches in a given land-use type, calculated as follows [75]:

(10) $$D_{i}=\frac{Q_{i}+M_{i}}{4}+\frac{Li}{2},$$

where *Q_i_* is the number of sample squares/total number of squares in plaque *i*, *M_i_* is the number of plaque *i*/total number of plaques, and *L_i_* is the area of plaque *i*/total area of quadrat.

According to relevant references and previous studies, the importance from high to low included landscape fragmentation (*C_i_*), landscape separation (*N_i_*), and landscape dominance (*D_i_*), with weights of 0.5, 0.3, and 0.2 assigned to the three indicators *a*, *b*, and *c*, respectively. [28,65,66].

The spatial distribution of the ecological risk index was analyzed through the natural breakpoint classification. The ecological risk index was divided into five categories: lowest risk (ERI ≤ 0.1), low risk (0.1 ≤ ERI ≤ 0.15), medium risk (0.15 ≤ ERI ≤ 0.35), high risk (0.35 ≤ ERI ≤ 0.45), and highest risk (ERI > 0.45).

### 3.5. Spatial Autocorrelation Analysis

Spatial autocorrelation analysis is used to present the spatial correlation features of the spatial reference unit and adjacent unit as a function of the attribute values (ERI in the study). Global spatial and local spatial autocorrelation analyses were performed by using the GeoDa software to describe the spatial distribution of landscape ecological risks in Zhangjiachuan County. The Moran index (Moran’s I) was applied to measure the global spatial autocorrelation of the ecological risk, and the numerical values represent the clustered distribution, discrete distribution, and random distribution of landscape ecological risk in space [76]. Moran’s I can be calculated according to the following formula [77]:

(11) $$I=\frac{\sum_{i=1}^{n}\sum_{i=1}^{m}w_{ij}(x_{i}-\bar{x})(x_{j}-\bar{x)}}{S^{2}\sum_{i=1}^{n}\sum_{i=1}^{m}W_{ij}},$$

(12) $$S^{2}=\frac{1}{n}\sum_{i=1}^{n}{\left(x_{i}-x\right)}^{2},$$

(13) $$\bar{x}=\frac{1}{n}\sum_{i=1}^{n}x_{i},$$

where *x_i_* and *x_j_* represent the ERI of the reference cell *i* and the adjacent cell *j,* respectively, *n* and *m* represent the number of cells *i* and *j*, respectively, and *W_ij_* is a binary matrix of adjacent spaces. When region *i* is adjacent to region *j*, *W_ij_* is equal to the value 1; otherwise, *W_ij_* is equal to the value 0. *S*^2^ denotes the mean variance (*i* = 1, 2, …, *n*; *j* = 1, 2, …, *m*).

The local indicators of spatial autocorrelation (LISA) index reflects the degree to which a geographical phenomenon (or attribute values of a local unit in the entire region) is related to the same phenomenon or attribute values of adjacent local units. In general, Moran’s I terms are usually decomposed and represented on different area units to form a LISA cluster diagram. The LISA cluster maps are then analyzed to generate high–high aggregation “hotspots” and low–low aggregation “cold spots” in the local space to evaluate any abnormal local spatial feature [78,79]. The formula for calculating the LISA index is as follows:

(14) $$LISA=z_{i}\sum_{j=1}^{n}w_{ij}z_{ij}(i\neq j),$$

where *z_i_* and *z_j_* represent the standardization of ERI in cells *i* and *j*, respectively, and *w_i_* is a spatial weight matrix. If *I_i_* > 0, the spatial difference between cell *i* and its neighboring cell *j* is very small, with a high–high cluster (HH, highest value in a high value neighborhoods) or low–low cluster (LL, lowest value in a low value neighborhood); if *I_i_* < 0, there is a significant spatial difference in ecological risk, with high–low outliers (HL, high values in low value neighborhoods) and low–high outliers (LH, low values in high-value neighborhoods) [80,81].

## 4. Results

### 4.1. Spatiotemporal Characteristics of Land-Use Changes

**Figure 5:**
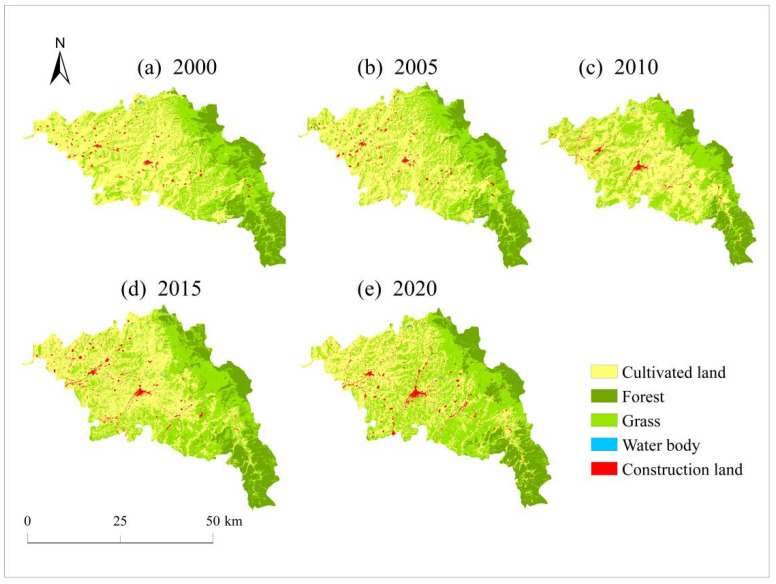
Spatial distribution of land use in Zhangjiachuan County from 2000 to 2020.

The land types used in Zhangjiachuan County include cultivated land, forest, grass, water body, and construction land (Figure 5). In 2000, cultivated land was the most widely distributed land-use type in Zhangjiachuan County, with an area accounting for 48.76% of the total area, followed by grass and forest, accounting for 33.45% and 16.58%, respectively. The construction land and water bodies accounted for 1.15% and 0.06%, respectively. Cultivated land, construction land, and grass are widely distributed throughout the central and western parts of Zhangjiachuan County, while forests are mainly distributed in the east.

From 2000 to 2020, the land-use changes in Zhangjiachuan County were mainly characterized by a decrease in cultivated land areas and an increase in grass and construction land areas. The proportions of areas with water or forest changed less. From 2000 to 2020, the cultivated land areas in Zhangjiachuan County decreased by 35.22% (164.66 km^2^), areas with construction land increased by 35.23% (10.86 km^2^), and grass areas increased by 14.66% (74.50 km^2^). In turn, areas with forest or water body changed relatively little, increasing only 6.52% (14.99 km^2^) and 10.96% (0.25 km^2^), respectively (Table 3). Among the different time periods, the cultivated land areas decreased the most from 2000 to 2005, decreasing by 13.76% (76.48 km^2^). Areas with grass saw the largest increase rate from 2015 to 2020, up by 8.26% (42 km^2^), while those with construction land increased the most from 2010 to 2015, increasing by 18.07% (5.20 km^2^).

**Figure 6:**
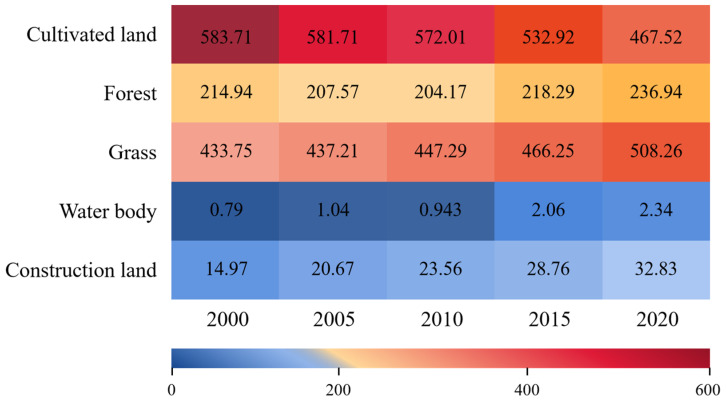
Area of various land use types in Zhangjiachuan County from 2000 to 2020 (km^2^).

From 2000 to 2020, the areas with cultivated land constantly decreased in Zhangjiachuan County, prompting the expansion of construction land and grass areas. From 2000 to 2005, around 173.62 km^2^ of cultivated land was converted for different land uses, of which 94.14% was converted into grass and 4.17% was converted into construction land. The transferred area of grass was 193.19 km^2^, 84.61% of which came from cultivated land, and the transferred area of construction land was 10.61 km^2^, 68.22% of which came from cultivated land. From 2005 to 2010, the transferred area of cultivated land was 168.37 km^2^, of which 89.09% was converted into grass and 8.16% was converted into construction land. The transferred area of grass was 191.67 km^2^, 78.61% of which was formerly cultivated land, while the transferred area of construction lands amounted to 18.72 km^2^, 73.38% of which was previously cultivated land. From 2010 to 2015, the area of cultivated land transferred out was 180.18 km^2^, of which 85.78% was converted into grass and 6.93% was converted into construction land. The transferred area of grass amounted to 194.53 km^2^, 79.45% of which came from cultivated land, and the transferred area of construction land was 19.43 km^2^, 64.29% of which previously consisted of cultivated land. From 2015 to 2020, the area of cultivated land transferred out was 181.94 km^2^, of which 87.70% was converted into grass and 9.04% was converted into construction land. The transferred area of grass was 189.41 km^2^, 84.24% of which formerly consisted of cultivated land, and the transferred area of construction land was 20.55 km^2^, with 80.04% having previously consisted of cultivated land (Figure 6).

### 4.2. Spatiotemporal Changes of Landscape Ecological Risks

**Figure 7:**
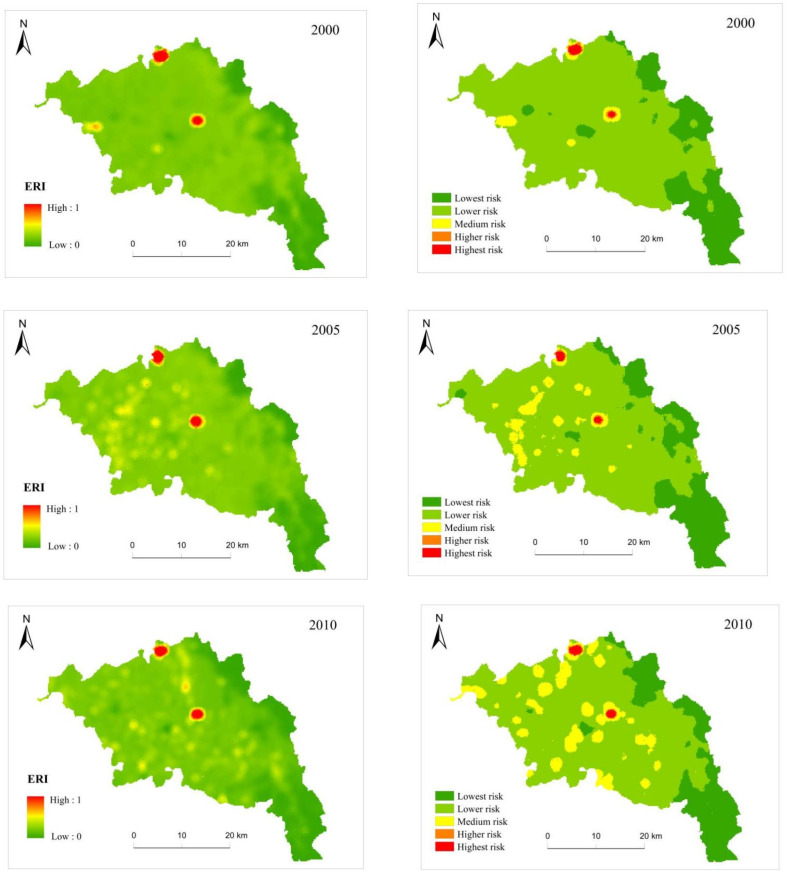

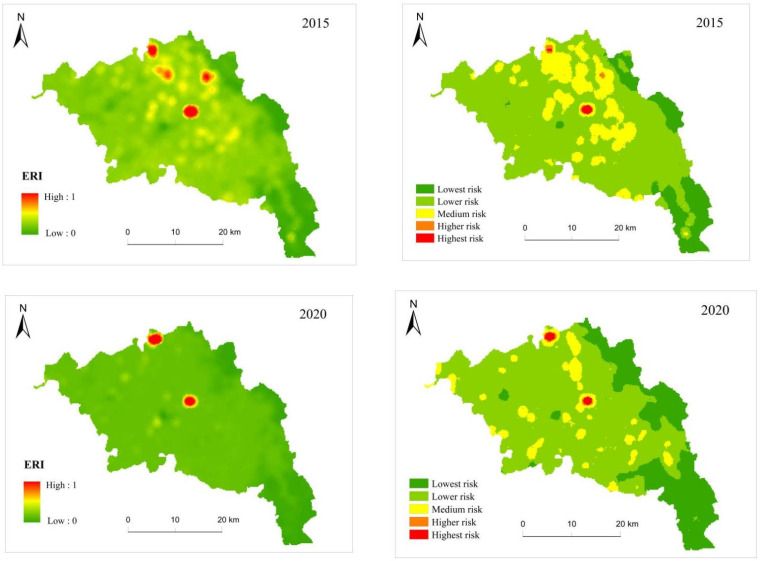
Spatial distribution of ecological risk index (**left**) and grade (**right**) in Zhangjiachuan County from 2000 to 2020.

In 2000, the landscape ecological risk area of Zhangjiachuan County was mainly concentrated near the Shixiakou Reservoir in the north and the Dongxiakou Reservoir in the middle; the low-risk area was mainly concentrated in the Longshan mountainous area in the east. On the whole, the ecological risk of land-use changes in Zhangjiachuan County presented a trend of being high in the west to low in the east but being mainly low (Figure 7). From 2000 to 2020, the ecological risks of land-use changes in Zhangjiachuan County were mainly the transformation between low risk and medium risk, with the lowest-risk area changing very little. From 2000 to 2005, the moderate-risk-level regions gradually emerged in the central and western regions of Zhangjiachuan County, and the low-risk areas basically remained unchanged. From 2005 to 2010, the change pattern of medium-risk regions was similar to that of the previous stage, further expanding in the central and western regions. From 2010 to 2015, the medium-risk areas further increased, the geographic center was shifted back to the midwest from the west of the previous stage, and the risk area distribution was relatively concentrated. Lastly, from 2015 to 2020, the ecological risk in Zhangjiachuan County improved, and the overall pattern was basically the same as that of the previous stage, with the areas prone to medium-level risks decreasing.

**Figure 8:**
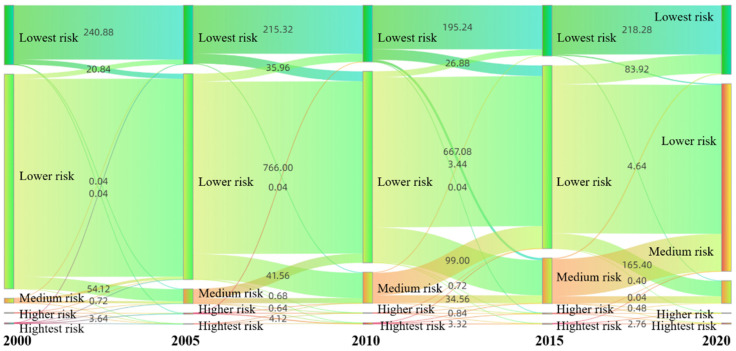
Ecological risk transfer in Zhangjiachuan County from 2000 to 2020 (km^2^).

From 2000 to 2020, the ecological risk value of each ecological risk cell increased and decreased, and the overall ecological risk value showed a trend of increasing first and decreasing later (Figure 8). However, the lowest-risk areas and low-risk areas showed a trend of decreasing and then increasing, while the areas of medium, high, and highest risk first increased and then decreased. From 2000 to 2015, the areas of the lowest risk and low risk levels decreased by 17.14% (38.76 km^2^) and 17.22% (140.40 km^2^), respectively; the areas of medium risk, high risk, and highest risk increased by 89.53% (181.2 km^2^), 21.50% (0.92 km^2^), and 11.27% (0.64 km^2^), respectively. The ecological risks associated with land-use changes increased during this period. From 2015 to 2020, the areas associated with the lowest risk and low risk levels were expanded by 26.42% (81.24 km^2^) and 2.27% (18.92 km^2^), and the areas of medium risk, high risk, and highest risk decreased by 67.49% (81.56 km^2^), 59.70% (1.6 km^2^), and 32.63% (1.24 km^2^), respectively. The ecological risks of land-use changes decreased during this period.

### 4.3. Spatial Autocorrelation Analysis

**Figure 9:**
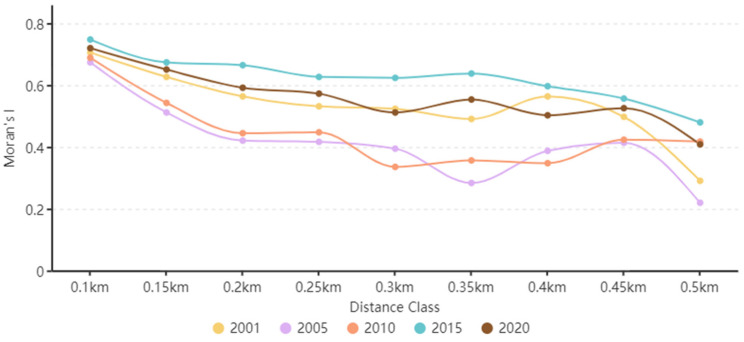
Moran’s I values for the ecological risk index (ERI) using different distances.

The spatial autocorrelation of the ecological risks of land-use changes is closely related to spatial gain. Figure 9 shows the calculated results of Moran’s I at different spatial distances varying from 0.1 to 0.5 km. All Moran’s I values were greater than 0, indicating that the ecological risk values of adjacent cells were similar in space, and the ecological risk index of Zhangjiachuan County had a significant positive spatial correlation within 20 years (*p* < 0.05). Moran’s I with a space distance of 0.1 km was the largest and decreased with increasing distance. When the distance ranged from 0.1 to 0.15 km, Moran’s I showed consistent change trends in 2000, 2005, 2010, 2015, and 2020. When equal distance Moran’s I values were considered, the value was the lowest in 2005 and the highest in 2015, showing a general change trend of decline from 2000 to 2005, increase from 2005 to 2015, and decline from 2015 to 2020. This suggests that the spatial concentration of ecological over the studied time period decreased slightly from 2000 to 2005, increased gradually from 2005 to 2015, and decreased slightly from 2015 to 2020.

**Figure 10:**
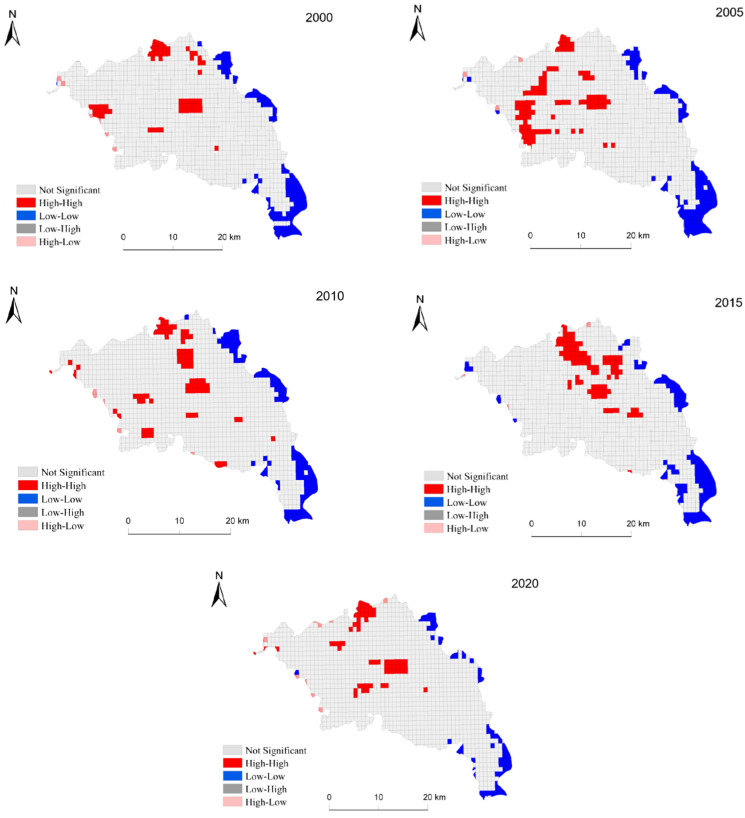
Local indicators of spatial autocorrelation (LISA) map of the local spatial auto-orrelation of ecological risks in Zhangjiachuan County from 2000 to 2020.

The Moran index can often be used to study the overall distribution and spatial aggregation of a region, but it cannot show the spatial correlations within it; therefore, local autocorrelation LISA analysis was used to research the correlation degree of ecological risks in the study area and to study whether it exhibits some spatial aggregation (Figure 10). Four kinds of significant autocorrelations—high–high (HH), low–low (LL), low–high (LH), and high–low (HL)—appeared in the study area. The HH areas were mainly found in the center and northwest of Zhangjiachuan County, the LL areas were mainly distributed in the east and southeast, and the HL areas mainly occurred in the northwest and southwest. The ecological risks in the midwestern regions were relatively high, while those in the east and southeast regions were relatively low.

### 4.4. Verification of the Theoretical Framework of Ecological Risk Transformation of Land-Use Change

**Figure 11:**
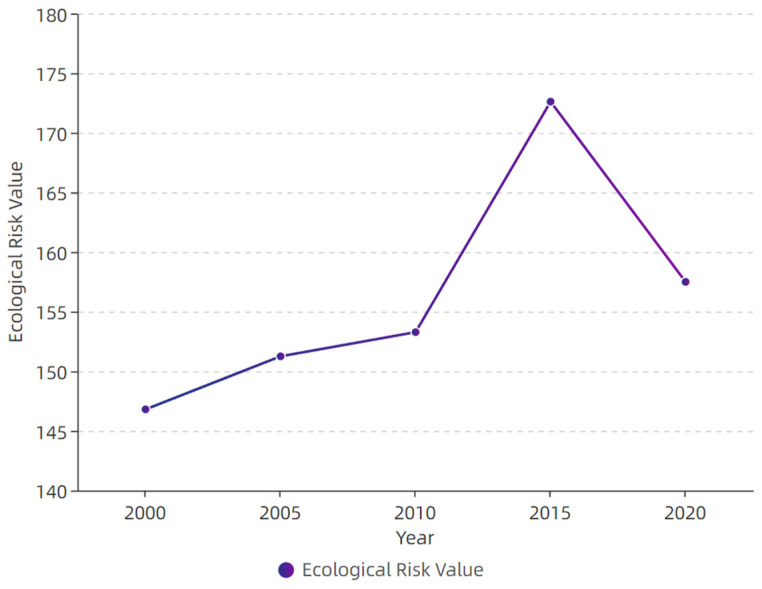
Overall landscape ecological risk values of Zhangjiachuan County from 2000 to 2020.

In general, the ecological risk index of land-use changes in Zhangjiachuan County showed a trend of increasing first and then decreasing (Figure 11). Over 2000–2010, the change was relatively slow. The risk index increased from 146.8 in 2000 to 153.3 in 2010 by a total increase of 4.2% (6.5). Over 2010–2015, the ecological risk value increased sharply to 172.6 in 2015, a total increase of 11.2% (19.3). From 2015 to 2020, the ecological risk value decreased to 157.5 in 2020, with a reduction rate of 8.7% (15.1). The value of the ecological risk index showed an “inverted U-shaped” change trend in the area over the 20 studied years. Therefore, the temporal and spatial change characteristics of ecological risks of land-use changes in Zhangjiachuan County are consistent with the ecological risk change trend of the transformation theoretical framework of ecological risks of land-use changes.

## 5. Discussion

### 5.1. Formation Mechanism of the Spatial Differentiation of Ecological Risks

Land is the carrier of the main social and economic activities, and it is one of the most intuitive forms of human development and utilization of the natural geographical environment [82]. Changes in the structures and patterns of lands are strongly related to the spatial and temporal distribution and dynamics of landscape ecological risks. According to the landscape ecological risk assessment of spatial patterns and analyses of the influence of the number, functions, and combinations of landscape elements on ecological risk, land-use changes can affect the structure and function of landscapes to varying degrees [11]. The ecological risks of land-use changes in Zhangjiachuan County are high in the west and low in the east, and the overall risk is low. Longshan Mountain is located In the east of the study area, with high terrains, as well as a cold and humid climate, consisting mostly of forested lands with a low human impact; the ecological risk in the east is, therefore, low. The midwestern regions are characterized by frequent droughts, large surface water runoff, rare vegetation, and serious water and soil loss; most areas consists of cultivated and construction lands with intense human influences, thereby making the ecological risk in this region high. The areas associated with the most serious risks were located close to the Shixiakou Reservoir in the north and Dongxiakou Reservoir in the middle. The construction of the reservoir has changed the original flood plain of the river, damaged the habitat of organisms, flooded the land vegetation, and reduced the extent of the wetland areas, greatly changing the type of land use and triggering ecological risks.

The ecological risks of land-use changes in Zhangjiachuan County generally showed a trend of increasing first and then decreasing. The risk increased from 2000 to 2015 and decreased slightly from 2015 to 2020. The west of Zhangjiachuan County is located in the hilly and gully area of the Loess Plateau, with a low terrain, poor vegetation, and the abandonment of cultivated lands; increased landscape fragmentation was observed from 2000 to 2005. At the same time, the expansion of Longshan Town in the west also introduced certain risks to the ecology of the studied area. Over 2005–2010, the main reason for the expansion of medium-risk areas in the midwestern regions was that the expansion of construction land occupied cultivated land, destroyed the landscape connectivity of the original cultivated lands, and deteriorated the landscape ecology. From 2010 to 2015, the implementation of the transformation project of medium- and low-yield fields in the comprehensive agricultural development of the Dayang Township in the west improved the ecological problems in the west of Zhangjiachuan County. However, due to the influence of national policies, some farmers in Zhangjiachuan County migrated to Xinjiang and other places, which aggravated the loss of cultivated land areas in the east. At the same time, the town of Zhangjiachuan expanded rapidly, which made the landscape more fragmented and dramatically increased the ecological risks in the area. In general, from 2000 to 2015, on the one hand, Zhangjiachuan County had a complex geographical environment, undulating terrain, changeable climate, and frequent natural disasters, resulting in certain ecological risks. On the other hand, the remoteness of the area and difficulties associated with transportation both hindered the improvement of agricultural mechanization; therefore, the area could not meet the requirements of high-intensity farming processes [83]. At the same time, the economic benefits of agricultural planting were not obvious, which led to the low enthusiasm of some farmers for planting, along with the growing phenomenon of large areas of idle cultivated land, turning cultivated land into wasteland [84,85]. At the same time, with the expansion of the population and the improvement of the per capita consumption level, the continuous development and expansion of construction lands led to a more severe loss and fragmentation of cultivated lands, and frequent human activities and urbanization fragmented and complicated the landscape, thereby increasing the ecological risk.

With the rapid development of modern society and the economy from 2015 to 2020, increasingly serious ecological problems have attracted great attention from the state, and a series of deployments have been made; for example, the land greening action was launched to promote the comprehensive control of desertification, rocky desertification, and soil erosion. During the 13th Five-Year Plan period, Zhangjiachuan County planned and implemented four small watershed comprehensive treatment projects, including the 2016 National Comprehensive Agricultural Development Project, Zhangjiachuan Maguan Project, and Area Comprehensive Control Project, which further promoted the comprehensive control level of soil erosion and ecological quality of Zhangjiachuan County [86]. Therefore, from 2015 to 2020, the ecological risk decreased as a result of the abovementioned measures.

According to the theoretical framework of ecological risk transformation of land-use changes and a field verification of Zhangjiachuan County, the construction land areas in Zhangjiachuan County began to expand on a small scale during the early stages of the study, occupying part of the cultivated lands and grasslands; at the same time, a phenomenon of abandoned cultivated lands also prevailed, which damaged the landscape connectivity, resulting in a relatively slow increase of ecological risks from 2000 to 2010. From 2010 to 2015, Zhangjiachuan Town continued to expand outward, construction land started to emerge in a disorderly manner, and the loss of cultivated land intensified, leading to a sharp increase in ecological risks during this period. The implementation of the ecological environment governance project improved the ecological quality of the area and reduced the ecological risks over 2015–2020. Therefore, the overall ecological risk of land-use changes in Zhangjiachuan County from 2000 to 2020 presented an “inverted U-shaped” change trend consistent with the theoretical framework of the ecological risk transformation of land-use changes.

### 5.2. Comparison of the Ecological Risk Results and Other Studies

This study put forward a theoretical framework for the transformation of the ecological risks of land-use changes. It is believed that there was a transformation of the ecological risk of land-use changes in Zhangjiachuan County, showing an “inverted U-shaped” change. The relationship between land-use changes and ecological risks is complex. According to the forest transformation theory and an environmental Kuznets curve, this paper further improved the research process and content of the ecological risk of land-use changes. It provides an important theoretical reference for ascertaining the ecological risks associated with the processes of regional land-use transformation, expanding the research vision of regional ecological risks and forming a new perspective. At the same time, it upholds the concept of theory guiding practice, fills the shortcomings of the relative lack of previous research theories in this field, and provides theoretical support for the ecological risk research of land-use changes in other regions worldwide.

The reasons of the ecological risk of land-use change in this study area are described below. On the one hand, Zhangjiachuan County is experiencing ecological problems due to changes in its geographical environment and climate. On the other hand, strong human activities have caused the loss of cultivated lands and expansion of construction lands, leading to a more fragmented landscape and increasing the intensity of the ecological risks in the area. The relationship between land-use changes and landscape ecological risk is complex, and different modes of action can cause different degrees of ecological risk. Song et al. [87] studied the impact of land-use changes in mining areas on the ecological environment and showed that the land use/coverage of the mining area often underwent great changes with changes in mining activities, while the vegetation, soil, and water would also suffer different degrees of damage. Kayumba [88] studied the wetlands in Bayanbulk and found that human activities such as urbanization, overgrazing, and tourism seriously changed the ecosystem of the region, causing ecological deterioration and ecological risks.

According to the analysis of the landscape ecological risks of land-use changes in Zhangjiachuan County, intense human activities have sharply reduced the natural vegetation in the area, and the landscape has become more fragmented, resulting in a series of ecological problems and increasing the associated ecological risks. However, these risks are reversible. The ecological problems could be remedied through certain countermeasures, thereby reducing the ecological risks. This is consistent with the research conclusions of Liang [67] on the dynamic changes of land use and ecological risks in the Three Gorges Reservoir Area of China and those of Liu [13] on the Shaanxi Province of China. However, the observations made in this work differ from the research conclusions of Peng [11] on the ecological risks of the Yongjiang River basin in Zhejiang Province, those of Yan [27] on typical areas of the Yellow River basin in China, and those of Hassan Omar [3] who studied land0use changes in Zanzibar (Tanzania). The main reason for this difference is the certain blindness in carrying out ecological protection and restoration in some areas, with a lack of systematic and comprehensive arrangements, resulting in few ecological restoration effects. Therefore, different land-use control strategies should be developed to handle ecological problems in different regions, and ecological restoration interventions should be conducted to further realize the sustainable utilization of land resources.

### 5.3. Policy Enlightenment

Although the problem of cultivated land fragmentation still existed in the study area from 2015 to 2020, and the areas of construction lands continued to expand, and Zhangjiachuan County effectively improved the landscape ecology of the area by conducting a series of comprehensive management projects. This paper showed that the implementation of governance projects can improve the regional ecology of a region and plays a positive role in reducing ecological risks. For example, following the completion of a dam in 2009, the Three Gorges Reservoir Area implemented a number of measures such as a project of returning cultivated lands to forests and developing orchards to promote the management of the Three Gorges Reservoir area and reduce ecological risks. Moreover, the ecological water transmission project in the Tarim River basin has increased the natural vegetation area and the riverbank vegetation coverage area, and the overall natural ecology of the whole region has been improved [89]. At present, the ecological governance projects have achieved some results, but the factors that cause ecological risks still exist and have not been effectively addressed. Therefore, Zhangjiachuan County still faces a certain degree of ecological risks in the future. According to our results, future policy formulations should focus on the aspects described below.

First, the ecological restoration of the reservoir should be strengthened. On the one hand, the protection of the ecological environment around the reservoir should be enhanced. We should promote the cultivation of water conservation forests, protect the existing forests and grasslands in the reservoir area, follow appropriate measures to return cultivated lands to forests, effectively prevent water and soil loss, ensure water supplies, and purify and reduce water pollution. On the other hand, the water environment in the reservoir area should be comprehensively improved. By improving the county government infrastructure, we should increase the construction of sewage treatment systems, adjust agricultural production structures, improve planting methods, advocate for ecological agriculture, effectively control agricultural non-point-source pollution, and minimize the ecological risk of the reservoir.

Secondly, the agricultural operation mode should be innovated. On the one hand, rural land reforms should be promoted. To some extent, owning fragmentated cultivated lands is a key factor limiting the enthusiasm of farmers for production and leading to a low efficiency of cultivated land utilization. Under the background of the transfer of the rural labor force in Zhangjiachuan County, the rural land transfer system should be improved, the transfer behavior should be standardized, and cultivated lands should be centrally managed. At the same time, the land management department should strengthen the supervision and management of the transfer of land management rights to protect the interests of farmers. On the other hand, the mode of agricultural development should be changed. The development of the “party building + enterprises + cooperatives + production bases + farmers” should be explored and established, an industrial chain of “farmers planting + cooperative purchasing + enterprise production” should be formed, and a stable increase in the income of farmers should be ensured. At the same time, according to the climate, terrain, environment, land resources, and other characteristics of all townships (towns) and villages in the county, timely and reasonably adjustments should be made by planting different types of crops according to local conditions, selecting good varieties, vigorously promoting good varieties and advanced applicable technologies, increasing the economic benefits of agricultural planting, and improving the enthusiasm of farmers.

## 6. Conclusions

Using the forest transformation theory and an environmental Kuznets curve, this paper analyzed the close relationship between land-use changes and the ecological risks associated with these; the theoretical framework of the ecological risk transformation of land-use changes was considered for Zhangjiachuan County, Tianshui City, Gansu Province as an example. The spatial and temporal pattern changes of land use in Zhangjiachuan County from 2000 to 2020 were analyzed, and the spatial and temporal characteristics of landscape ecological risks in the study area were evaluated. Our results show that the land-use changes in Zhangjiachuan County from 2000 to 2020 mainly included a decrease in the area of cultivated lands and the expansion of grasslands and construction lands. The ecological risks increased slowly from 2000 to 2010, increased sharply from 2010 to 2015, and decreased from 2015 to 2020. The overall “inverted U-shaped” trend is consistent with the transformation theoretical framework of the ecological risks of land-use changes. In terms of patterns, the ecological risk of land-use changes in Zhangjiachuan County was basically reflected in the distribution trend of high in the west and low in the east. In terms of areas, those with the lowest risk and low risk levels decreased from 2000 to 2015, and areas with medium risk, high risk, and highest risk levels increased, thereby aggravating the ecological risks of the area. From 2015 to 2020, the areas with the lowest risk and low risk levels increased, while the areas with medium, high, and highest risk levels decreased, reducing the ecological risks during this period. By comparing the ecological risk change of land-use change with that of some countries, systematic and comprehensive arrangements should be made when carrying out regional ecological restoration projects, and differentiated land-use control strategies should be formulated according to local conditions to achieve the sustainable use of land resources.

There were some limitations to this study. The spatiotemporal evolution of ecological risks is a comprehensive and complex process, which is affected by the population, economy, and production, thus necessitating further research and analysis. At the same time, longer time series should be studied to clarify the impact of land-use changes on ecological risks, and the direct relationship between landscape patterns and ecological risks also requires further research. In summary, future ecological risk research on land-use changes should consider highly variable social, economic, and environmental factors. The relationship between land-use changes and ecological risks would also need to be confirmed through more empirical research. While ecological protection is not a linear decision-making process, it requires a dynamic adaptive response to changing land-use types. Land-use changes in areas with high ecological risks should be paid attention to, land remediation efforts should be multiplied, and the resilience to ecological risks should be improved.

## Figures and Tables

**Table 1:** Landsat image information.

Data Source	Landsat Image Set ID	Year
Landsat 5	LANDSAT/LT05/C01/T1_TOA	2000–2011
Landsat 7	LANDSAT/LE07/C01/T1_TOA	2012
Landsat 8	LANDSAT/LC08/C01/T1_TOA	2013–2020

**Table 2:** Auxiliary classification spectral index.

Spectral Index	Calculation Formula
MNDWI [54]	$MNDWI=\frac{\left(G-MIR\right)}{\left(G+MIR\right)}$
RVI [55]	$RVI=\frac{NIR}{R}$
DVI [56]	DVI=NIR−R
SAVI [57]	$SAVI=\frac{1.5*(NIR-R)}{\left(NIR+R+0.5\right)}$
NDMI [58]	$NDMI=\frac{\left(G-SWIR\right)}{\left(G+SWIR\right)}$
EVI [59]	$EVI=\frac{2.5*(NIR-R)}{\left(NIR+6*R-7.5*B+1\right)}$

Note: (MNDWI: modified normalized difference water index; RVI: ratio vegetation index; DVI: difference vegetable index; SAVI: soil-adjusted vegetation index; NDMI: normalized dry matter index; EVI: enhanced vegetation index).

**Table 3:** Land-use transfer matrix from 2000 to 2020.

km^2^	(Year) 2020					
2000		Cultivated Land	Forest	Grass	Water Body	Construction Land
	Cultivated land	345.79	8.78	209.33	1.01	18.05
	Forest	4.76	180.17	29.34	0.01	0.23
	Grass	109.18	47.48	267.11	0.77	8.74
	Water body	0.13	0.03	0.06	0.4	0.1
	Construction land	7.21	0.05	1.9	0.11	5.7

## Data Availability

All relevant datasets in this study are described in the manuscript.
